# 白细胞介素35的在肿瘤发展中的作用

**DOI:** 10.3779/j.issn.1009-3419.2016.04.09

**Published:** 2016-04-20

**Authors:** 崇标 黄, 野 田, 焱 崔, 杰 徐, 亮 辛, 雪玲 杨, 大亮 齐

**Affiliations:** 1 300060 天津，国家癌症临床研究中心，天津市肿瘤防治重点实验室，天津医科大学肿瘤医院高级病房 Department of Senior Ward, Tianjin Medical University Cancer Institute and Hospital, National Clinical Research Center for Cancer, Key Laboratory of Cancer Prevention and Therapy, Tianjin 300060, China; 2 300060 天津，国家癌症临床研究中心，天津市肿瘤防治重点实验室，介入治疗肿瘤科 Department of Interventional Treatment, Tianjin Medical University Cancer Institute and Hospital, National Clinical Research Center for Cancer, Key Laboratory of Cancer Prevention and Therapy, Tianjin 300060, China

**Keywords:** 白细胞介素, IL-35, 肿瘤, 肿瘤免疫, Interleukin, Interleukin-35, Tumor, Tumor immunology

## Abstract

白细胞介素（interleukin, IL）-35是IL-12细胞因子家族的新成员，自2007年发现以来IL-35很快成为了相关领域的研究热点。与IL-12家族的其他成员（IL-12、IL-23以及IL-27）相类似，IL-35也是由异源性的*α*链及β链组成，即p35和EBI3蛋白组成。目前的研究发现IL-35的功能主要表现在两个方面，一方面是IL-35在体内某些炎性疾病及自身免疫性疾病患者体内异常表达，IL-35在这些疾病的发生发展过程中起到重要作用。另一方面，IL-35在某些肿瘤中也表达异常，在肿瘤的发展过程中起到了重要作用。本文对IL-35的结构及信号通路进行了阐述，并总结了IL-35在肿瘤发展中的作用的最新研究进展。

## Abstract (en)

Interleukin(IL)-35 is a new member of the interleukin-12 superfamily. Since its first report in 2007, IL-35 rapidly became a research highlight in the field of immunology. Like other IL-12 superfamily members, IL-35 was a heterodimer which was composed of an *α* chain P35 and a β chain Epstein-Barr virus induced gene 3 (EBI3). Recent research work revealed two distinct roles of IL-35. Firstly, IL-35 is highly expressed in some kinds of inflammatory diseases and autoimmune diseases and plays import roles in the pathogenesis. Secondly, IL-35 is positively expressed in some cancers and plays some roles in the process of tumor progression. Here we demonstrate the structure and the signalling of IL-35. We reviewed the the roles of IL-35 in promoting tumor progression.

白细胞介素（interleukin, IL）是由多种细胞产生并作用于多种细胞的一类细胞因子^[1]^。由于最初发现其由白细胞产生并具有调节白细胞的生理功能，因此而得名^[2]^；后来的研究^[3, 4]^发现白细胞介素能影响包括肿瘤细胞在内的多种细胞的增殖、成熟、粘附及转移等多方面的生理过程。IL-35是IL-12家族的最新成员，由EBI3（Epstein-Barr virus induced gene 3）及P35亚基组成的异源性二聚体^[5]^。研究^[6, 7]^发现IL-35可由单核细胞、T细胞、B细胞、Treg细胞及肿瘤细胞等多种类型的细胞表达，通过激活Jak-STAT信号通路参与免疫功能的调节^[8]^，与转化生长因子β（transforming growth factor-β, TGF-β）和IL-10一样被认为是最重要的三种免疫抑制因子之一^[9]^。除了直接抑制肿瘤微环境的肿瘤免疫状态，引起免疫逃逸，IL-35还能直接作用于某些类型的肿瘤细胞促进肿瘤的进展^[10]^。本文将IL-35在肿瘤发展中的作用的研究进展进行综述。

## 1 IL-12家族概述

IL-12家族包括IL-12、IL-23、IL-27和IL-35四种白细胞介素。这四种白介素的结构相似，由不同的亚基组成^[11]^，包括一个独立表达的*α*亚基（P19、P28或P35）和一个β亚基（P40或EBI3）。与IL-6超家族类似，该*α*链包含四螺旋束结构，而β链包含可溶性Ⅰ类细胞因子受体链的结构特征，例如IL-6R*α*。IL-12是IL-12超家族最早发现的分子，IL-12是由p35和p40链组成的异源性二聚体，但是研究^[12]^发现细胞内p40亚单位的同源二聚体和单体都可以稳定地存在，并可以作为对IL-12的功能拮抗剂。IL-23是P19 *α*链和P40 β链形成的异源性二聚体^[13]^。虽然IL-23和IL-12对下游基因的调控作用不一样，但同IL-12一样也是促炎因子。抗原呈递细胞受微生物病原体刺激可产生IL-23，进而诱发的T细胞生成IL-12并具有促进Th1细胞的分化的作用。但在与IL-12不同的是，IL-23能诱导IL-17生成，诱导T细胞向Th17细胞分化^[14, 15]^。IL-27是P28 *α*链和EBI3 β链组成的异源性二聚体，同时具有促炎和诱导免疫抑制的双向免疫调节功能^[16]^。IL-35是由P35 *α*链的和EBI3 β链组成的异源性二聚体，从目前的研究结果来看IL-35具有严格的免疫抑制功能^[17]^，这与其他3种IL-12家族成员的作用完全不同。因此，IL-12家族里面仅IL-35具有作为一个抗自身免疫性疾病和自身炎症性疾病的细胞因子的潜能。

IL-12家族在肿瘤的发展及治疗中起到重要的作用。IL-12在诱导Th1型细胞的分化中发挥重要调节作用，通过促进IFN-*γ*的分泌使NK细胞和T细胞大量增殖并增强其细胞杀伤能力，在抗肿瘤免疫中扮演着重要的角色^[18]^。IL-23在多种肿瘤中表达且促进肿瘤的发生、转移及生长^[19]^。IL-27对肿瘤的作用正好相反，很多研究发现IL-27具有抗肿瘤生长的作用^[20, 21]^。IL-35是一种免疫抑制因子，可通过多种途径促进肿瘤的进展^[22]^。

2007年Collison及其同事在*Nature*上首先报道了IL-35的结构及其免疫抑制功能^[17]^。T细胞表面的Toll样受体TLR3、TLR4受IFN-*γ*等激动剂刺激后，开始分泌IL-35^[23]^。但与其他的IL-12家族成员不同的是，IL-35主要由CD4^+^ Foxp3^+^调节性T细胞（regulatoryTcells, Treg）产生^[17]^。另外，活化的B细胞或者活化的内皮细胞、单核细胞以及某些肿瘤细胞也能分泌IL-35^[22, 24]^。在人和小鼠体内，IL-35可诱导效应T细胞转变成为一类新型调节性T细胞，即iTr35细胞^[25]^。与其他IL-12家族不同的是，IL-35可以通过调节性T细胞IL-10的分泌抑制的CD4^+^效应T细胞、Th1和Th17的生成及增殖。有意思的是，与经典CD4^+^CD25^+^FoxP3^+^ Treg细胞相比，CD4^+^ HLA-G^+^的人胸腺源性调节性T细胞（tTreg）明显分泌更高的IL-35水平^[26]^。

将IL-35的两个亚基*EBI3*和*p35*基因的敲除小鼠模型中，Treg细胞的功能受到影响，这说明IL-35对于维持Treg细胞的功能具有重要的作用^[17]^。虽然p35或EBI3缺陷小鼠没有明显的自身免疫性疾病或炎性疾病，并且与普通的野生型小鼠相比，EBI3^-/-^和p35^-/-^小鼠的Treg细胞比例以及Foxp3表达水平没有明显差异，但是，EBI3^-/-^和p35^-/-^ Treg细胞的免疫抑制能力明显降低^[17]^。

与胸腺起源的天然Treg细胞（nTreg）不同，诱导型调节性T细胞（iTr细胞）主要由IL-10及TGF-β作用于CD4^+^ Foxp3^-^ T细胞转变而来^[27]^。IL-35能够抑制CD4^+^效应T细胞的增殖，也能够激活效应T细胞分泌IL-35（自激活）^[17]^，这提示IL-35具有诱导调节性T细胞的潜能。Collison等^[25]^于2010年在*Nature Immunology*上发表文章，报道了一群新的调节性T细胞，他们将能分泌IL-35的调节性T细胞定义为IL-35诱导性调节性T细胞（iTr35细胞）。该细胞群是Foxp3-调节性T细胞，其本质特征是能分泌IL-35，并且这种调节性T细胞的功能不依赖于Foxp3的表达。iTr35细胞仅能被IL-35诱导产生，不能经由其他已知的抑制性细胞因子如IL-10或者TGF-β诱导产生。因此，IL-35可能在介导感染性耐受或者肿瘤免疫耐受中起到了重要作用^[28]^。

由p35和EBI3的单链形成的重组IL-35融合蛋白能显著地抑制T细胞增殖和Th17细胞分化^[29]^。IL-35融合蛋白可增强CD8^+^CTLA-4^+^Treg细胞对自体T细胞的增殖的抑制活性^[30]^。体液免疫也显示出通过rIL-35诱导人B细胞转化成分泌IL-35以及IL-10的抑制性调节B细胞。

## 2 IL-35的信号通路

不同的IL-12家族成员采用不同的受体进行信号传导过程。IL-12和IL-23的受体共享IL-12Rβ1亚基但分别使用IL-12Rβ2和IL-23R亚基。IL-27的受体由糖蛋白130（Glycoprotein 130, gp130）和WSX-1（IL-27R）组成。而IL-35的受体由gp130亚基IL-12Rβ2亚基组成。但是，有意思的是，IL35也能通过gp130和IL-12Rβ2组成的gp130:gp130以及IL-12Rβ2: IL-12Rβ2这两种同源二聚体传导信号^[31]^。IL-12Rβ2主要由IFN-*α*和IFN-*γ*诱导的T细胞和自然杀伤T细胞表达，但是在幼稚T细胞不表达^[32]^。gp130广泛表达在几乎所有的器官中^[33]^。gp130是很多细胞因子参与信号转导的受体复合物组分之一，可能是发育过程中起关键作用的信号转导受体。gp130缺陷的小鼠在胚胎发育的时候就会死亡^[34]^，而IL-12Rβ2缺陷性小鼠更容易受到自身免疫和B细胞恶性肿瘤的侵扰^[35]^。在人类的各种免疫相关疾病，如原发性胆性肝硬化、克罗恩病、银屑病、全身性硬化症、1型糖尿病、特发性皮炎、白塞氏病以及Vogt-Koyanagi-Harada（VKH）综合征可能与IL-12Rβ2表达异常或者基因多态性相关^[29, 36-39]^。这些现象说明IL-35信号通路可能在保持机体免疫平衡、维护机体功能中起到重要作用。

IL-35通过与IL-12Rβ2和gp130组成的IL-12Rβ2:gp130异源性二聚体或IL-12Rβ2:IL-12Rβ2及gp130:gp130同源二聚体结合来传导信号^[25]^。IL-35受体激活后，继而通过激活Janus激酶（JAK）家族成员来进行信号转导。JAK磷酸化后进一步磷酸化STAT家族的STAT1和STAT4进行信号转导，入核后其结合于不同位点，促进p35和EBI3基因的启动子转录从而增加IL-35的表达。由于IL-12Rβ2及gp130受体亚基主要激活的JAK分子分别为JAK1及JAK2，分别磷酸化下游的STAT4及STAT1分子，因此当IL-35结合于其中的一种同源二聚体受体中时，他的信号传导途径只有一个分支被激活，即IL-12Rβ2:IL-12Rβ2主要激活JAK1-STAT4信号分支，而gp130:gp130主要激活JAK2-STAT1分支^[31, 40]^。虽然单支信号通路的激活也能引起免疫抑制现象，但是与通过全功能的IL-12Rβ2/gp130的异二聚体受体信号传导相比较其抑制能力明显降低^[31]^。

## 3 IL-35在肿瘤发展中的作用

### 3.1 IL-35在肿瘤微环境中重要的免疫抑制因子

虽然IL-35是最早在Treg中发现的免疫调节因子，在免疫调节中起到重要的功能。但是，随后有研究发现，多种肿瘤细胞也可表达IL-35，并且发现IL-35在肿瘤的发展中起到重要作用。Wang等^[22]^研究发现，IL-35在多种肿瘤组织及肿瘤细胞系中都有表达，例如B细胞淋巴瘤，鼻咽癌及黑色素瘤。为了研究肿瘤源性IL-35的作用，他们构建了过表达IL-35的浆细胞瘤J558和B16黑色素瘤细胞系。他们发现，在体外实验中IL-35并不能影响肿瘤细胞的增殖及存活能力，但在Rag1/2缺陷型小鼠体内似乎却能刺激肿瘤微环境中肿瘤发生。同时，肿瘤源性IL-35能促进CD11b^+^Gr1^+^髓源性细胞聚集，从而促进肿瘤的血管生成。在免疫正常小鼠体内，IL-35的表达使肿瘤所引起的自发性的细胞毒性淋巴细胞反应明显下降。IL-35并不能直接抑制肿瘤抗原特异性CD8^+^T细胞的激活、分化及效应功能。但是，IL-35处理肿瘤细胞后其gp130表达上调，同时对细胞毒性淋巴细胞的反应敏感性下降，促进肿瘤微环境的免疫抑制状态。Tao等^[41]^发现急性髓性白血病患者的IL-35表达水平明显升高，IL-35通过促进Treg扩增同时抑制CD4^+^CD25^-^效应T细胞的增殖引起肿瘤的免疫逃逸。

Zeng等^[42]^研究了IL-35对结直肠癌的进展及预后的影响。他们检测了包含肺癌、结直肠癌及肝癌等9种人肿瘤细胞系（A549、SW480、BEL-7402、HepG2、SiHa、HPAC、MDA-MB-231及MCF）中IL-35两个亚基的表达水平。结果显示IL-35的两个亚基p35及EBI3共表达于这几个肿瘤细胞系，而正常的细胞系表达很少。他们发现，当用外源性的IFN-*γ*及TNF-*α*刺激后，肿瘤细胞表达IL-35的量增加。在另一项关于IL-35在结肠癌中的表达及作用的研究中，研究者发现肿瘤组织IL-35的表达水平与肿瘤的恶性程度及临床分期呈正相关，外周血IL-35表达水平与外周血Treg细胞数量成明显的正相关，作者认为肿瘤源性IL-35可能通过招募Treg细胞到肿瘤微环境中来促进肿瘤的生长。有研究用小鼠模型探索了IL-35对肿瘤的影响。他们用中和抗体拮抗IL-35的EBI3亚基后，肿瘤的生长速度明显受到抑制；肿瘤局部浸润的肿瘤浸润性淋巴细胞显著增加，活化的CD8^+^细胞以及处于增殖期的淋巴细胞也明显增加。总结上述研究，IL-35在肿瘤微环境肿瘤免疫中起到重要的抑制作用，引起了肿瘤的免疫逃逸，促进了肿瘤的进展。

### 3.2 IL-35对肿瘤细胞的直接作用

Nicholl等^[10]^用免疫组织化学方法研究IL-35在胰腺癌中的表达。他们发现胰腺癌肿瘤组织异常过表达IL-35。他们在细胞实验中发现，IL-35可以上调cyclin、Bcyclin D及cdk4的表达，同时下调p27的表达从而促进肿瘤细胞增殖；通过上调Bcl-2的表达以及下调TRAILR1的表达抑制肿瘤细胞的凋亡，进而促进胰腺癌的进展。Liao及其同事^[43]^分析现已发表的研究结果并且构建了一个数学模型，通过该模型的分析他们发现IL-35可以促进肿瘤的生长及血管生成，应用抗IL-35药物可以降低肿瘤的生长速度。Fan等^[44]^采用免疫组化方法分析了胃癌肿瘤组织中的IL-35表达水平，他们发现IL-35高表达水平与较大的肿瘤体积及侵犯深度显著相关，IL-35的表达水平与Ki-67及Bcl-2表达水平显著相关，因此他们认为IL-35可能通过调节胃癌的增殖及凋亡影响肿瘤的进展。Tao等^[41]^发现急性髓性白血病患者的IL-35水平明显升高，除了能诱发肿瘤的免疫逃逸机制外，还能直接促进肿瘤的增殖及抑制肿瘤的凋亡。但是，也有学者报道了不同的研究结果。在一项由Long及其同事^[45]^完成的乳腺癌的研究中，IL-35被报导具有抑制肿瘤的特性。研究者证实IL-35在多种肿瘤细胞中高表达，并且TNF-*α*及IFN-*γ*可以促进肿瘤细胞分泌IL-35。他们构建了过表达IL-35的肿瘤细胞稳转细胞系并检测了IL-35对肿瘤细胞增殖及凋亡的影响。他们发现，过表达IL-35可以引起肿瘤细胞G_1_期停滞，抑制细胞的增殖，促进血清饥饿诱导的细胞凋亡。他们采用RT-PCR检测了过表达细胞及对照细胞的一些增殖及凋亡相关基因的mRNA表达变化，结果显示过表达IL-35可以引起*Fas*基因的表达上调及Cyclin D1、Survivin及Bcl-2基因表达水平的下调。总结上述研究，IL-35可直接作用于多种肿瘤细胞，影响肿瘤的增殖、凋亡及血管生成等生理功能，但是目前对于这些作用的具体作用方式及机制仍然没有阐述清楚，需要研究进一步跟进。

肿瘤细胞的侵袭和转移在肿瘤发展过程中起着重要作用，虽然有研究发现IL-35表达与肿瘤的转移有相关性^[46, 47]^，但是并没有明确的证据证明IL-35能影响肿瘤的转移与侵袭。免疫抑制状态也是促进肿瘤转移的一个重要因素，鉴于IL-35是很强的免疫抑制因子，IL-35也可能通过免疫抑制来促肿瘤转移，但这需要进一步研究证实。

## 4 血浆IL-35在肿瘤诊断及预后中的意义

Jin等^[48]^探索了胰腺癌患者血浆中IL-35的水平及Treg细胞的关系。他们发现，与正常人对照人群相比较，胰腺癌患者血浆中IL-35的水平明显上升[(134.53±92.45) pg/mL *vs* (14.26±6.56) pg/mL]。同时他们也发现IL-35 mRNA的水平与IL-35蛋白水平正相关。他们进一步发现IL-35表达水平与淋巴结大小及临床分期成正相关。Gu等^[49]^用酶联免疫吸附测定（enzyme linked immunosorbent assay, ELISA）方法分析了106例非小细胞肺癌患者（non-small cell lung cancer, NSCLC）及78例正常对照的血浆标本中的IL-35表达水平，研究者发现NSCLC患者血浆IL-35水平较正常人明显升高 [(21.37±11.55) (pg/mL *vs* (10.09±5.32) pg/mL, *P* < 0.001)]，他们进一步发现高血浆IL-35表达水平与较差的TNM分期（*P* < 0.001）及淋巴结受累显著相关（*P* < 0.000, 1）；IL-35高表达水平与NSCLC患者的预后呈显著负相关，单因素及多因素分析发现血浆IL-35是NSCLC患者的独立预后因素。一项在结直肠癌中的研究^[42]^发现，结直肠癌患者血浆中的IL-35水平也明显升高，并且其表达水平与疾病的严重程度及肿瘤的临床分期相关；他们进一步分析发现，结直肠癌患者术后IL-35较术前明显升高，因此他们推测IL-35血浆水平与结直肠患者的不良预后相关。Tao等^[41]^发现急性髓性白血病患者外周血血浆中IL-35水平明显升高，并且其表达水平与疾病的临床分期显著相关。但是，在一项关于甲状腺癌的研究中却得到了相反的结论。Lu等^[50]^分析了甲状腺癌及甲状腺瘤患者血浆IL-35表达差异性，他们发现甲状腺癌患者血浆中的IL-35较甲状腺腺瘤患者的明显降低[(48.8±7.8) pg/ml *vs* (62.3±9.6) pg/ml; *P* < 0.05]，可作为甲状腺癌的诊断标志物，其受试者工作特征曲线（receiver operating characteristic curve, ROC）下面积为0.872, 2。总结上述研究，IL-35在多种肿瘤患者的血浆中表达水平升高，具有一定的肿瘤诊断意义及预测预后意义，可作为潜在的肿瘤标志物。

## 5 展望

综上所述，IL-35在多种肿瘤细胞中表达，其血浆表达水平具有一定的肿瘤诊断及预测预后意义，可作为潜在的肿瘤标志物。除了引起肿瘤微环境免疫抑制状态，导致肿瘤免疫逃逸外，IL-35可能能直接作用于肿瘤细胞本身，通过影响肿瘤细胞的增殖、凋亡及肿瘤的血管生成过程而影响肿瘤的进程。我们可以推断，阻断IL-35信号传递过程可能有助于抗肿瘤治疗。
